# Physical and Economic Impacts of Sea-Level Rise and Low Probability Flooding Events on Coastal Communities

**DOI:** 10.1371/journal.pone.0117030

**Published:** 2015-02-24

**Authors:** Thomas Prime, Jennifer M. Brown, Andrew J. Plater

**Affiliations:** 1 University of Liverpool, Environmental Science, Liverpool, Merseyside, United Kingdom; 2 National Oceanography Centre, Liverpool, Merseyside, United Kingdom; 3 University of Liverpool, Geography, Liverpool, Merseyside, United Kingdom; University of New England, AUSTRALIA

## Abstract

Conventionally flood mapping typically includes only a static water level (e.g. peak of a storm tide) in coastal flood inundation events. Additional factors become increasingly important when increased water-level thresholds are met during the combination of a storm tide and increased mean sea level. This research incorporates factors such as wave overtopping and river flow in a range of flood inundation scenarios of future sea-level projections for a UK case study of Fleetwood, northwest England. With increasing mean sea level it is shown that wave overtopping and river forcing have an important bearing on the cost of coastal flood events. The method presented converts inundation maps into monetary cost. This research demonstrates that under scenarios of joint extreme surge-wave-river events the cost of flooding can be increased by up to a factor of 8 compared with an increase in extent of up to a factor of 3 relative to “surge alone” event. This is due to different areas being exposed to different flood hazards and areas with common hazard where flood waters combine non-linearly. This shows that relying simply on flood extent and volume can under-predict the actual economic impact felt by a coastal community. Additionally, the scenario inundation depths have been presented as “brick course” maps, which represent a new way of interpreting flood maps. This is primarily aimed at stakeholders to increase levels of engagement within the coastal community.

## Introduction

### The significance of coastal flooding and storm surges

It is estimated that 46 million people were affected by flooding globally in 1990 [1], this number is set to increase due to increasing mean sea levels. If 1m of sea-level rise (SLR) occurs by 2100 with no other change e.g. resilience measures, it is estimated that this number would rise to 60 million people [2].

Examples of the devastating impacts of coastal flood events include the impacts of Hurricane Katrina, which caused 1570 deaths in Louisiana and caused $40–50 billion dollars of monetary losses [3]. It is predicted that it will take up to 8–11 years to reconstruct the urban infrastructure and environment [3]. More recently Hurricane Sandy produced a catastrophic storm surge which among other areas hit a section of the US coastline stretching from New Jersey to Rhode Island which contributed to a damage cost in excess of $50 billion dollars and 72 direct fatalities [4]. Storm surges in the Bay of Bengal have caused widespread flooding and loss of life, for example the Bhola Cyclone in 1970.

The UK is also susceptible to coastal flooding. A classic historical example is the North Sea storm surge that occurred in 1953 causing devastation in the UK and continental Europe, with the loss of over 1800 lives in the Netherlands [5] and 300 deaths in east and southeast England [6]. This proved something of a ‘tipping point’ in coastal planning, in response to this event sea walls were repaired and raised to increase the resilience of the country to coastal flooding. The Delta Plan in the Netherlands was established and led to construction of barriers across several of its estuaries over the next decades [7]. In 1953 many people were not aware of their vulnerability to storm surge flooding and received no warning, meaning that they were unprepared for the floodwater. Lack of communications inhibited the warning of other areas that flooding was imminent and prevented the request of external assistance [8]. Since 1953 there have been other storm surges with similar water elevation but these have had a lesser impact due to the building of coastal defences [9]. From this point on we use the term “storm tide” to consider the combined tidal and surge water level, the total high water level posing as the potential flood risk and hence threat to life and infrastructure.

In the UK it is estimated that there are approximately six million UK properties (one in six of all properties) currently exposed to some degree of flood risk, with 600,000 properties in areas of significant risk [10]. The UK floods in 2007 were estimated to have cost businesses £740 million; this is on average around £100,000 per affected business, with some taking 27 weeks to return to normal. Through appropriate risk management it is possible to become more resilient to these flooding events and gain economic benefit. It has been estimated for Europe that every £1 spent on adaptation and resilience measures represents 4 times its value in potential damage avoided [11]. The government has planned to spend £2.42 billion on flood and coastal erosion risk management between April 2011 to March 2015 [10]. From the 4 to 1 ratio mentioned above, this should provide £9.68 billion in benefits to the UK economy. However the impact of flooding is still felt; over the period 23/12/2013 to 28/02/2014 flooding has so far resulted in 18,700 insurance claims at a cost of £451 million [12].

### Impacts of Increased Mean Sea Level

Assuming no adaptation to increasing coastal flood risk, the expected annual damage (EAD) in England and Wales due to coastal flooding is predicted to increase [13]. Present-day EAD to residential and non-residential properties is of the order of £1.3 billion for the UK as a whole [10]. For residential properties alone the expected annual damage from tidal and river flooding in England and Wales is projected to increase from £640 million currently to over £1.1 billion by the 2020’s under a Medium UK Climate Projections 2009 (UKCP09) emissions scenario [11]. There are a range of SLR projections that are available for coastal flood risk assessment. The Intergovernmental Panel on Climate Change (IPCC) have recently updated their global projections of SLR which range from 28 to 98 cm by 2100 [14]. Focusing on the UK, planning and environmental management practitioners refer to the UKCP09 projections [15]. Under the medium emissions scenario these have a range of 0.21 m to 0.68 m in London by 2095, which is based on earlier IPCC sea-level rise projections, with their maximum plausible scenario H++ being 1.9 m [15]. Beyond 2100 there is less published data, however Jevrejeva et al. (2012) have projected values up to 2500AD, with values ranging from 0.13 m to 11.51 m depending on confidence level and projected climate scenario [16].

Projections of future SLR values are very uncertain, with continued concern that large increases in the 21^st^ century are possible and cannot be ruled out [17]. One of the biggest sources of uncertainty is the response of the large ice sheets on Antarctica and Greenland. For a temperature rise of 4°C or more by 2100 a SLR between 0.5 m and 2 m is estimated [17]. The probability of SLR occurring that is at the higher end of these projections is low but unquantifiable. However, a SLR of this magnitude would have a huge impact on the globe with up to 2.4% of the global population being displaced [17]. SLR needs to be monitored closely to detect any accelerations in the rate of rise, and climate-induced processes need to be better understood [17].

The impact of coastal storms is increasing due to SLR increasing the frequency of extreme water levels which will increase the occurrence and impact of storm surges [18] and waves [19]. Even a small increase of mean sea-level (MSL) can change the return period of an extreme water level. e.g. a SLR of 0.28 m will turn a 1 in 250 year extreme water level to a 1 in 50 year extreme water level in the area of study for this paper [20].

This is also true of other environmental events; the increase of MSL alters not only the extreme wave heights but also the frequency of occurrence of extreme wave conditions [21]. In addition to SLR there are many variables that effect coastal flooding; these range from a changing climate (i.e. possible increases in the occurrence and strength of storms) to coastal defence degradation. [22,23]. Future increases in storminess are assumed to not be a significant factor [15]. In addition to surge and SLR significant factors rarely considered in coastal flood risk analysis are wave overtopping and high river flow. In modelling studies these processes require multiple models to be coupled together [24]. If either of these events occurs over the same period as a storm tide then they can significantly affect the flood extent and depths of inundation. The combination of river and surge inundation is known to be non-linear [25].

SLR is again important for other flood factors as the elevated water level enables other processes to breach coastal sea defences. With increased MSL, t a certain threshold will be reached when wave overtopping will become a significant hazard due to the increased frequency of extreme sea level events. [26]. This is also true of river flows as higher MSL means that rivers are more likely to break their banks along tidal reaches and flood estuarine communities. Knowing these tipping points for sea level is important for long-term adaptive coastal management as it identifies possible future state changes within a system and when a change in planning strategy may be required.

### Coastal Inundation Modelling

There are many different types of flood inundation model, one dimensional (1D), two dimensional (2D), coupled 1 and 2 dimensional (1D/2D) and finally 3 dimensional (3D) models. 1D models are widely used to model fluvial hydraulics using 1D finite difference solutions of the Saint-Venant equations [27]. 1D models are unable to resolve complex floodplain flow fields and require post-processing to produce realistic flood extents. 2D models can achieve this and allow the complex interaction of channel and floodplain flow fields to be simulated. Such modelling is now more feasible through improvements in computational resources and affordability. This is important when trying to model urban flooding, meaning that recent urban flood modelling efforts have been focussed on coupling a 1D model with a 2D model. This is where a 1D river channel model is complimented by a 2D model of the floodplain. Examples of this type of model are LISFLOOD-FP, Delft-FLS, SOBEK1D2D and MIKE FLOOD [28]. However, 2D models are unable to model structural elements that may produce super-critical flows [28]. Finally the last type of model is a 3D model, an example of this is Delft3D which is a coupled 2D/3D model that can be used for investigating hydrodynamics, sediment transport, morphology and water quality [28].

Some of the above models are able to simulate tidal forcing, however most modelling approaches do not include wave overtopping. This is important as wave overtopping can have a big impact on flood extent as events that do not overtop flood defences in 2D simulations could still experience flooding due to wave overtopping [29]. This paper develops an accessible methodology that couples a 2D storage cell flood inundation model with a 1D shallow water Boussinesq wave model which allows the effect of wave overtopping sea defences to be added to a storm tide flood simulation.

Other forms of flooding or defence failure could also be considered, these include still water overflow and sea defence breaches. Neither of these are covered by this method, as still water overflow would require higher extreme sea levels beyond the scope of this paper. Breach inundation modelling could be undertaken for the area but it would require knowledge of the age and condition of the defences as this will be strongly correlated with likely defence failures. These are areas of future work as breaches in defences can be incorporated into the flood model and timed to occur at a defined point during the simulation.

### Addition of River and Wave forcing

Being able to combine a storm tide and river flow with wave overtopping allows a more complete picture of the risk and hazard a coastal community faces. For example, the impacts are under-estimated using a 2D raster model in isolation. However, the inundation model used here (LISFLOOD-FP [30]) is flexible enough to allow the incorporation of rivers and wave overtopping. A river flow hydrograph can be assigned to a section of the model boundary falling across a river mouth to simulate the river inflow, while wave overtopping flow rates for chosen extreme wave heights and period at varying water elevations can be set as inflows at the top of the sea defences to simulate wave overtopping within the model domain.

This study aims to identify the importance of wave overtopping and river flow, when calculated as monetary costs, in coastal flood scenarios with increased MSL.

### Inundation Scenarios

**Fig 1:**
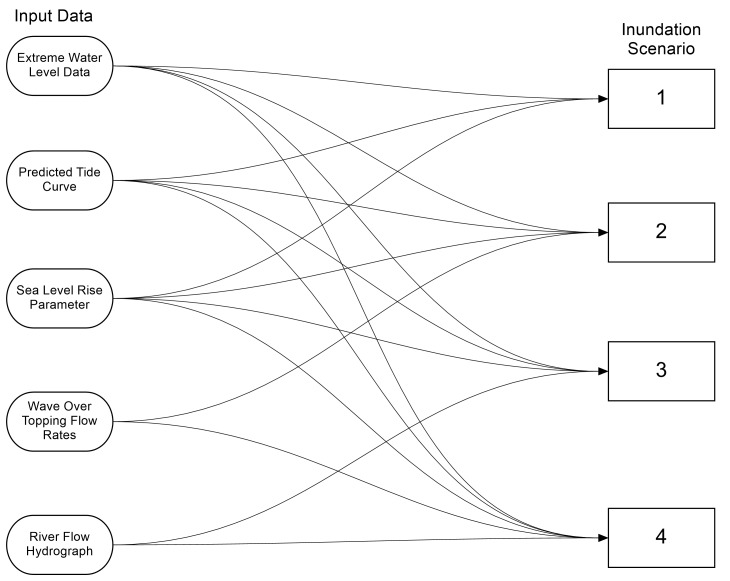
Flow chart showing the input data used for each of the four scenarios.

Fig. 1 shows the data inputs required for each of the proposed inundation scenarios. Section 3 details the data availability and how it is used in the scenarios. Some data are directly used within the process, e.g. the extreme water level data, river hydrograph etc., and some required derivation via pre-processing or model simulation, for example wave overtopping rates and the SLR parameter used.

### Site Selection

**Fig 2:**
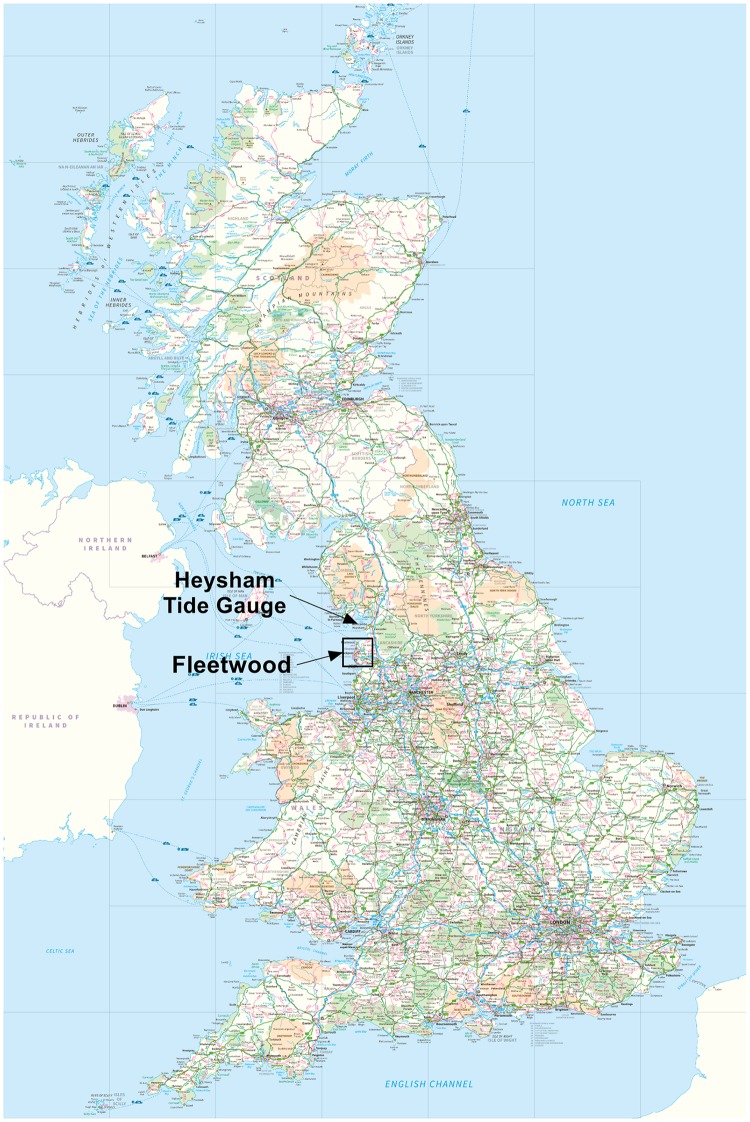
Map of the UK showing where Fleetwood and the tide gauge at Heysham are located [53].

The site that has been selected to trial this methodology is the Fleetwood coastline in Lancashire, northwest England, UK. Its location in relation to the rest of the UK is shown in Fig. 2. This figure also shows the location of the tide gauge at Heysham used for tidal prediction to generate the time-varying water elevation boundary curves. Historically Fleetwood is extremely susceptible to flooding, with coastal flooding occurring in 1927 with 6 deaths and again in 1977 when 1,800 properties were flooded, which led to the current concrete sea walls being constructed [31]. Both the 1927 and 1977 flooding was caused by storm surges during the 9th to 12th November 1977; as recorded at Heysham, the surges were 1.8 m high and occurred one to two hours earlier than the peak of a high spring tide [32].

Recently in the UK there have been a cluster of events that have caused major damage and disruption [33]. During a storm surge that occurred on 5th December 2013 there was a minimal amount of flooding at this location, this was due to spray being blown over the sea defences by the high winds. The concrete walls built after the 1977 flood successfully prevented any wave overtopping. At Heysham the surge height was 2.15 m, which is a larger surge than in 1977, showing that the current resilience of Fleetwood is significantly greater than it was before the flooding in November 1977. The return period for the total water level at the case study site was 1 in 75 years [20], and the Blackpool wave rider buoy data [34] for the same period demonstrates that the wave height and period were found to have a return period in excess of 1000 years, when compared with 140-year UKCP09 model wave data [35]. The highest recorded water elevation that occurred at Heysham during the 5^th^ December surge was 6.15 m above Ordnance Datum (AOD). The scenarios with increased mean sea levels that are explored in this paper are at a higher value than this with an increase of approximately 0.85 m. This takes into account a longer return period (1 in 250 rather than 1 in 75) and a potential SLR parameter (0.65 m).

**Fig 3:**
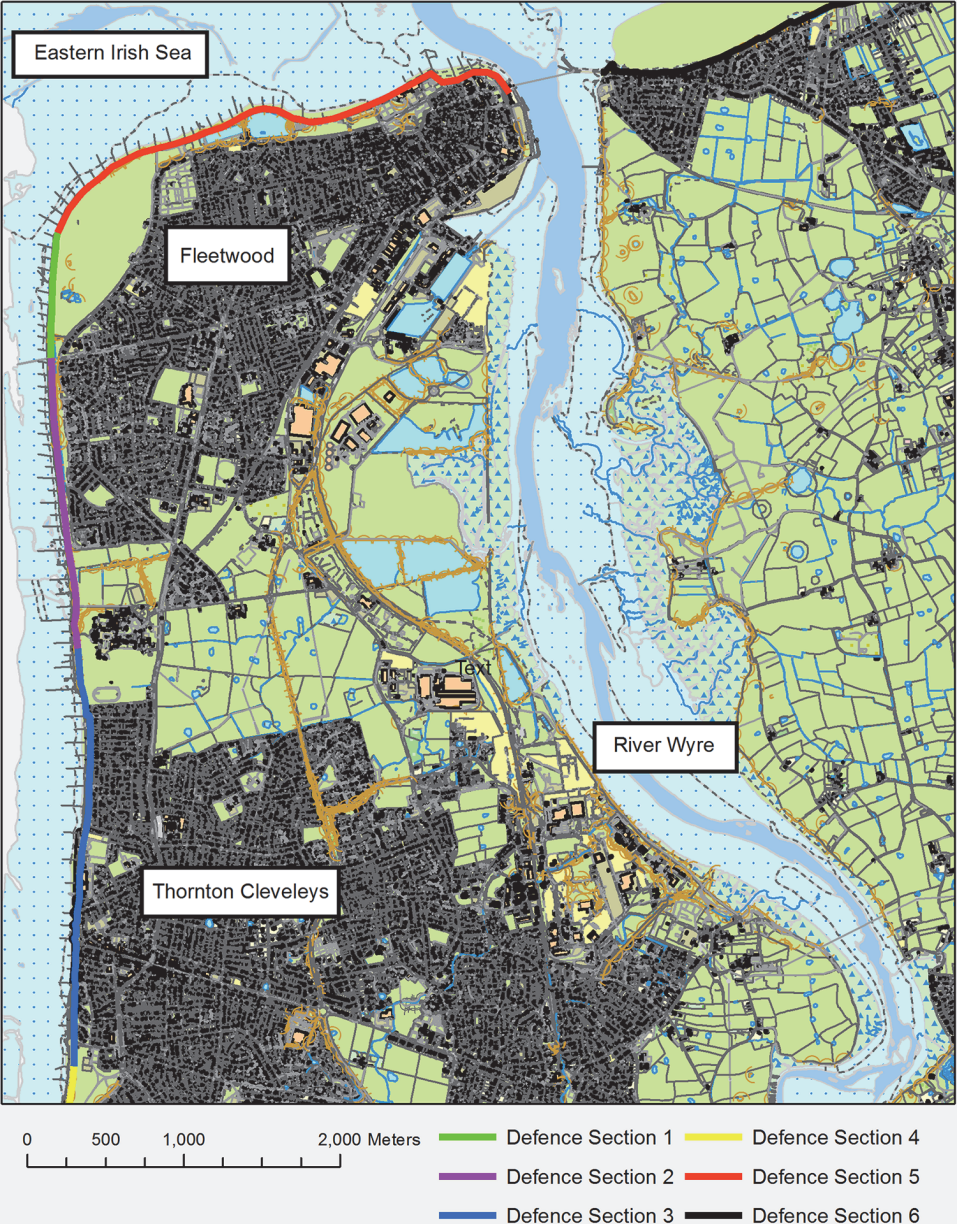
Detailed view of the model domain, each of the defence sections detailed in Table 1 are shown in varying colours.

The model domain (Fig. 3) is 9 km by 8.3 km and is centered on the areas of Fleetwood and Thornton-Cleveleys, with parts of Anchorsholme to the south. This is an area of low-lying land vulnerable to wave and river flooding, with extensive sea defences that have recently been refurbished. The model domain also contains the River Wyre. The protection provided by the sea defence ranges from a 1 in 500 year extreme water level (EWL) down to a 1 in 20 year EWL [36]. The area to the west of the Wyre is predominantly urban residential housing, with some industrial sites close to the river. To the east of the Wyre the area is predominantly rural, with arable land being prominent, along with some urban areas close to the coast in the north. The area is of interest to coastal managers to determine the resilience of the area to future sea-level projections.

While Fleetwood has sea defences to protect it, as demonstrated by the minimal flooding caused by the surge on 5th December 2013, it is vulnerable to SLR. Small amounts of SLR will significantly reduce the return period of the water level during storms and surges that pose a flood risk [20]. A 1 in 250 year EWL was selected as it relates to the return periods used in shoreline management plans and coastal defence management. After a review of the coastal defence data provided by the Environment Agency (EA), on average most sea defences protecting Fleetwood are designed to cope with a 1 in 200 year EWL.

For the purposes of this study, the sea defences at Fleetwood have been divided into 6 sections (Fig. 3); these sections were identified using a coastal defence database [36]. A significant change in defence crest height defines where a new defence section begins and ends, details of these are listed in Table 1.

**Table 1:** Details of sea defences for Fleetwood coastline.

Section	Crest Height (metres)	Defence Details along section
**1**	11	Concrete and cobble aprons with recurve wall
**2**	8.3–7.8	Concrete apron area with re curved wall
		Mass concrete wall with promenade
**3**	7.7–10	Seawall with stepped revetment
		Mass concrete wall with rear splash wall
		Concrete and masonry aprons
**4**	7.7	Seawall
**5**	6.9–8.1	Aprons with wall and promenade to rear
		Concrete aprons with small wall and promenade
		Beach ridge with promenade
		Concrete slab revetment
		Concrete and masonry walls
**6**	7–7.6	Embankment and splash wall
		Embankment and channel flood wall

## Methods

**Fig 4:**
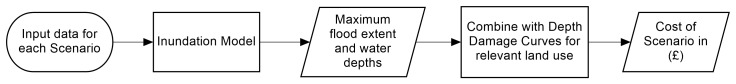
Flow chart showing the process followed to derive the cost of each scenario.

Fig. 4 shows a simple flow chart that details the steps taken from taking the input data, and getting the economic cost of the scenario at the end. Fig. 1 shows which input data is required for each scenario, essentially the tide, surge and SLR inputs remain the same for each scenario, and the only changing factors are the wave overtopping and river flow inputs. Section 1 goes through each of the input data shown in this flow chart (Fig. 1). Sections 2 to 3 detail the additional steps shown in Fig. 4. Finally sections 4 and 5, which are not described by the flow charts, provide additional methodology by explaining the sensitivity analysis that was performed, as well as additional model parameters required by the inundation model.

### 1 Input data

#### 1.1 Extreme water level data and Predicted Tide Curve

The EA has provided a geospatial file that has extreme water heights at 16 different return periods every 2 km along the UK coastline [20]. The 1 in 250 year exceedance values were used in this instance. Every data point that was within the model domain was used to define the extreme water level elevation at the model boundary closest to each data point.

**Fig 5:**
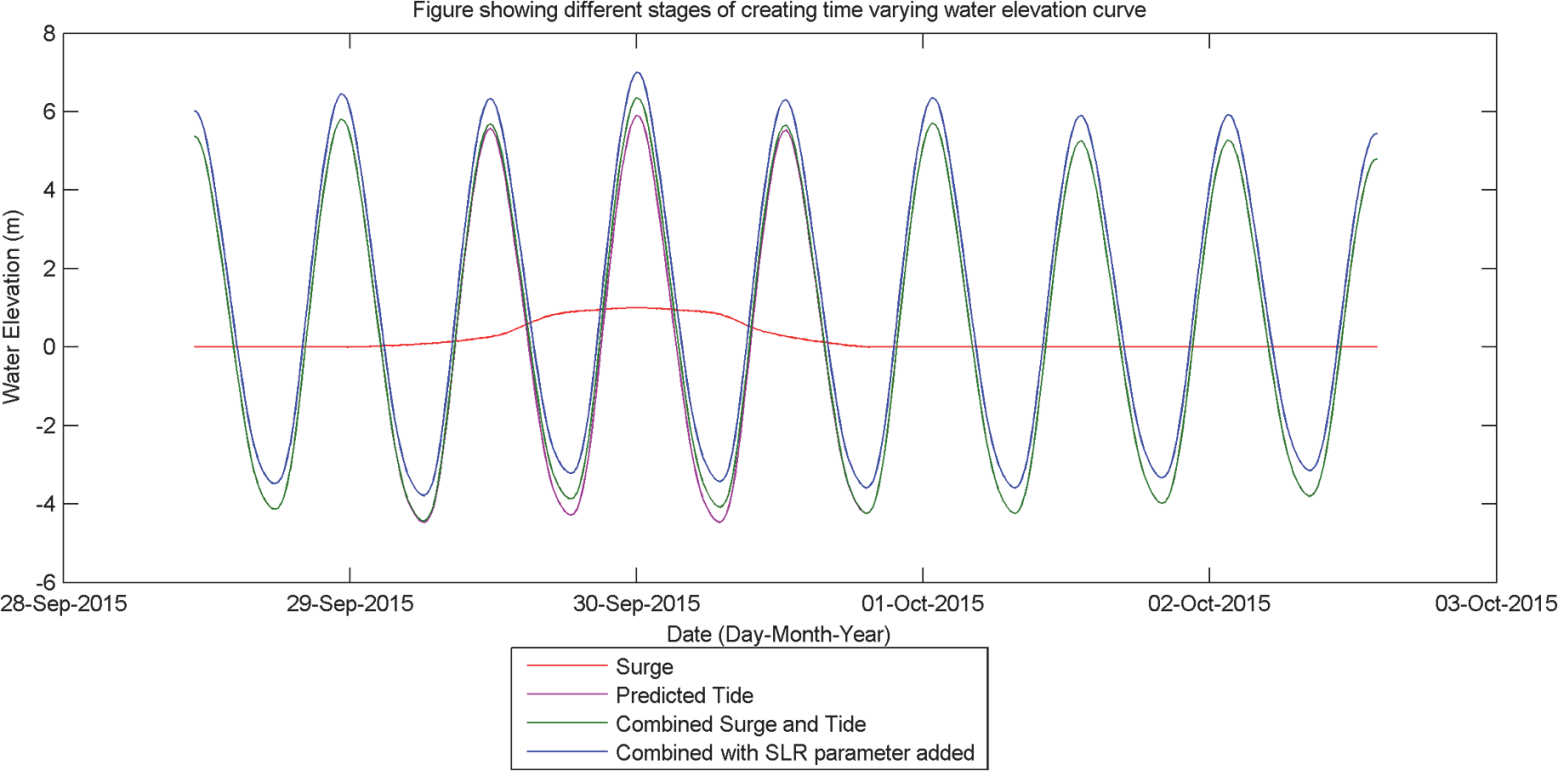
Graph showing the different stages in generating the time varying water elevation curve used at the model boundary [20].

To turn these extreme water level elevations into a time varying surge component, the EA have also provided representative surge curves for various tide gauges around the UK [20]. The closest one to Fleetwood is Heysham which is approximately 17 km north along the coast. The surge curve varies from 0 to 1 with 1 being the peak of the surge and 0 being no surge, for a simulation time of 100 hrs (red curve Fig. 5).

The final piece of data required is a predicted tidal curve; this was generated using a piece of software called POLTIPS3 which is available from the national tide sea level facility. The period selected was the 30/09/2015 when a highest astronomical tide is predicted. 15-minute interval data for several days either side of this date were generated and overlaid on to the surge curve, lining up the tide and surge peaks (purple curve Fig. 5).

The difference between the maximum predicted tidal high water (30^th^ September 2015, Fig. 5) and the selected extreme water level to be simulated is then calculated. This value is then multiplied by the 0 to 1 values of the surge curve to create the surge elevation over the possible 100 hr storm period, the peak surge occurring at the predicted tidal high water. The resulting surge elevation is then added to the predicted tidal curve giving a storm tide curve that peaks at the extreme water level selected (green curve Fig. 5).

Finally the SLR parameter (calculated in section 1.2) is added to all values to produce the desired target time-varying water elevation. This process was performed on each of the EA data points within the model domain and the resulting time-varying water elevation curve was applied to the relevant sections of the offshore model boundary (blue curve Fig. 5).

#### 1.2 Sea-Level Rise Parameter

**Fig 6:**
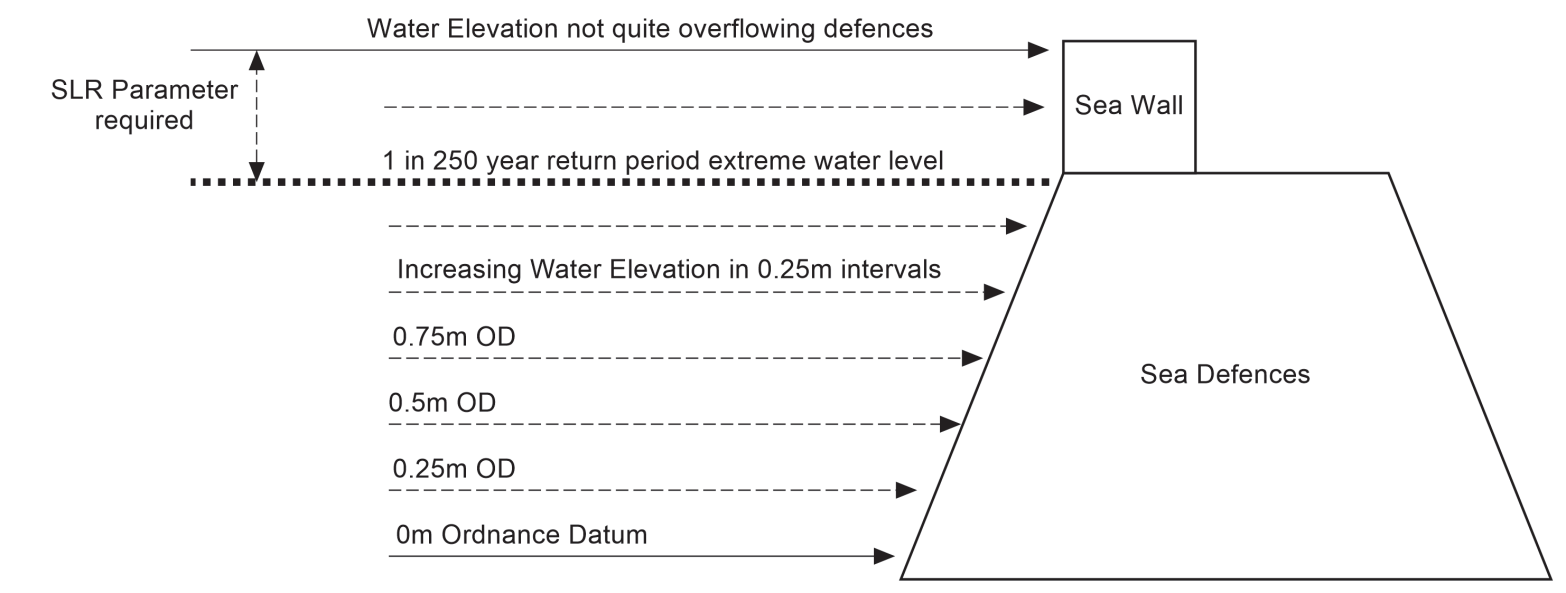
Diagram showing how the SLR parameter was selected.

To simulate the worst case scenario, a SLR parameter has to be selected, one that will just stop slightly short of overflowing current sea defences in still water flood simulations. This will show the biggest impact that wave overtopping and high river flows could potentially have. To identify this parameter level a simple inundation model simulation was run that has a time-varying water level at the boundary, this started at 0 m OD and was then increased by incremental steps of 0.25 m until the still water level overflowed the defences. The water level before this point was selected as the target water elevation that the potential future storm tide would need to achieve. Fig. 6 shows this schematically, the SLR parameter is the difference between this target water elevation and the current 1 in 250 year extreme water level elevation. This SLR value of 0.65 m has been identified as the tipping point for significantly increased flood risk for the present-day defences in this region. Selecting the SLR parameter in this way makes it an arbitrary figure, one that is not linked to any probabilistic SLR projections. However the value is within the UKCP09 relative sea-level rise projections by 2100 [15]. From closer inspection this value is just below the 0.68 m that makes up the upper range value from 1990 to 2095 for London (being a relative sea-level rise projection this value also takes into account vertical land movement). The parameter selected is also well under the 1.9 m plausible maximum of the H++ scenario provided by UKCP09 for 2100 [15].

#### 1.3 Wave overtopping flow rates

The Shallow Water Boussinesq Model (SWAB) is a semi-implicit shallow water Boussinesq model that has been developed to account for random wave breaking, impact and overtopping of sea walls [37]. This model has been tested against field data for overtopping at Blackpool and Anchorsholme, approximately 11 km south of Fleetwood. It has also been compared with 1:15 scale wave flume tests. Overall the overtopping output from this model has been found to agree well with field and flume measurements [37].

**Fig 7:**
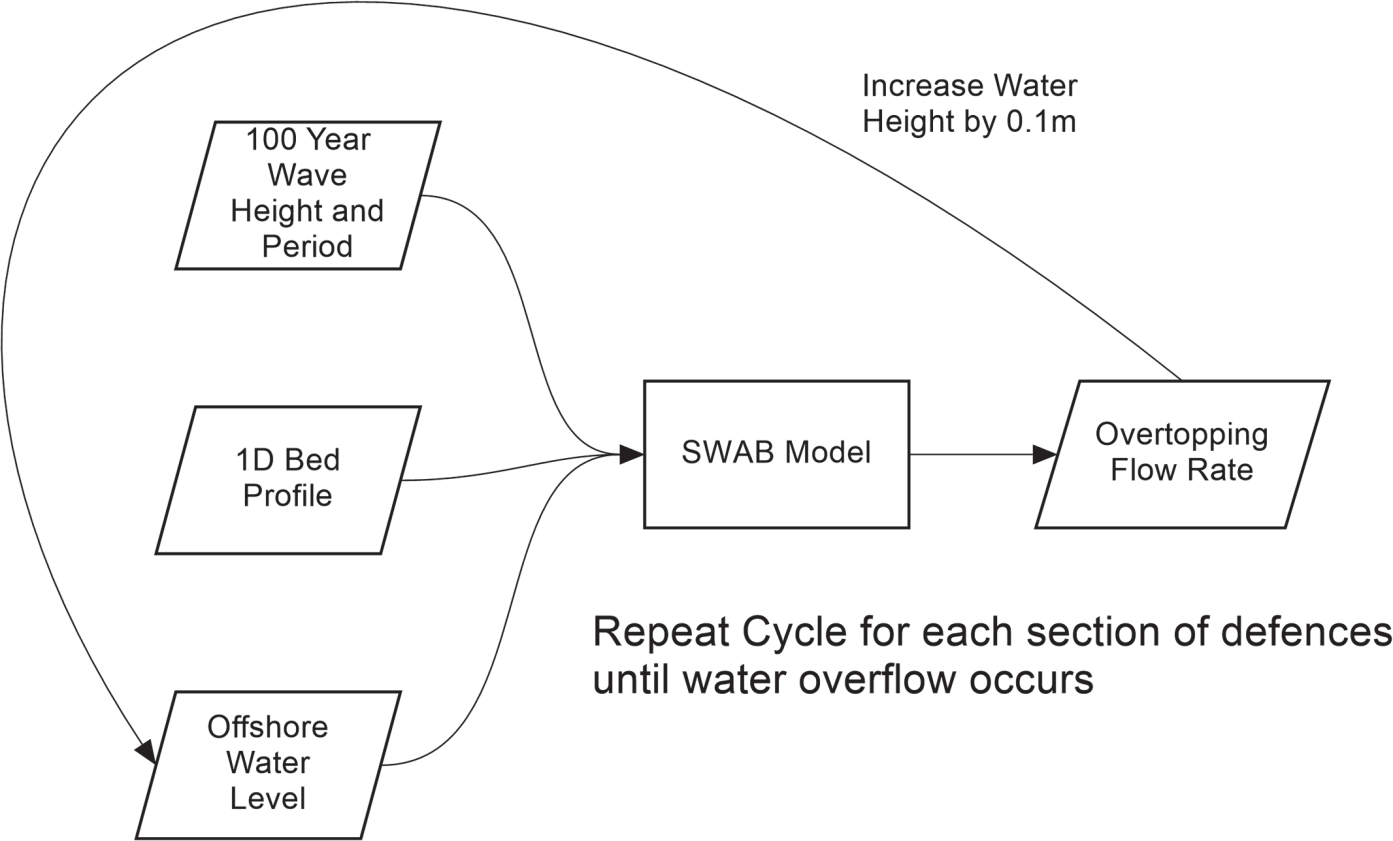
Flow chart showing the process of generating overtopping flow rates.

Here, the freely available online SWAB interface has been used to derive overtopping rates for various wave height return periods. This was achieved by extracting the full wave height and period data from UKCP09 datasets for the closest relevant location. Return period analysis was then performed on the wave height and peak periods to calculate the wave height and period exceedance values for 10, 100 and 1000 years. The method used to derive overtopping rates with SWAB is detailed in Fig. 7, this flow chart shows the steps made to calculate overtopping rates at 0.1m intervals for the relevant sections of sea defences. The defences have been split into sections (Fig. 3) and a 1D transect was extracted from the EA airborne laser altimetry (LiDAR) [38] which comprises the sea bed, sea defences and the land behind. This transect lies along the most common wave direction for the defence location identified using the UKCP09 data [15]. This transect provides the bed profile for the 1D SWAB model, which provides details of the bathymetry fronting the sea defences, the height and shape of the defences and the topography of the floodplain behind. To calculate overtopping, the model starts from a low water elevation value—one that does not cause any overtopping. The model is then run with a selected wave height and period, in this case the 1 in 100 year exceedance value. The model simulates a wave spectrum using the wave height and period inputs. This process is repeated with increased still water heights at 0.1 m intervals at the SWAB model boundary until the water overflows the sea defences. The overtopping rates calculated from the volume overtopped can then have a relationship to water level that allows the time-varying still water height at the boundary of the model to be associated with an overtopping rate.

**Fig 8:**
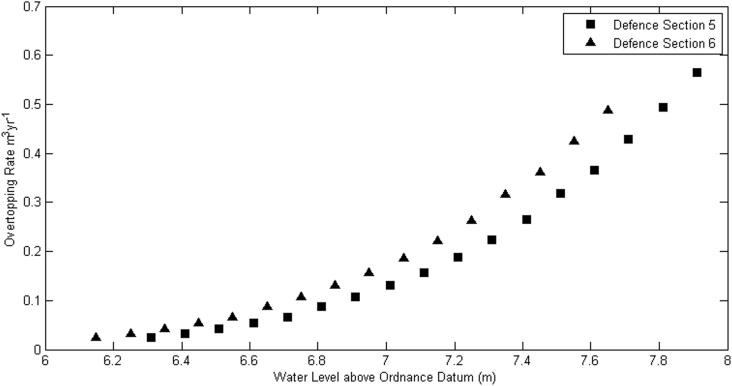
Overtopping rates derived by the SWAB model for Defence sections 5 (squares) and section 6 (triangles) during the water elevations of the 1 in 250 year storm tide.

This overtopping rate is added into the model domain as a point source of water at any relevant cells, in this case at 5 m intervals (every grid cell) along the top of the seawall defences, to provide the time-varying overtopping during a (in this case 1 in 100 year) event. Repeating this for all of the defence sections it was found that with a sea-level rise parameter of 0.65 m and a storm tide return period of 1 in 250 years only two sets of the defences overflow (Sections 5 and 6 highlighted in Fig. 3). Fig. 8 shows the output flow rates for the SWAB model for both section 5 and 6, of the northern defences during the 1 in 250 year storm tide.

#### 1.4 River flow hydrograph

**Fig 9:**
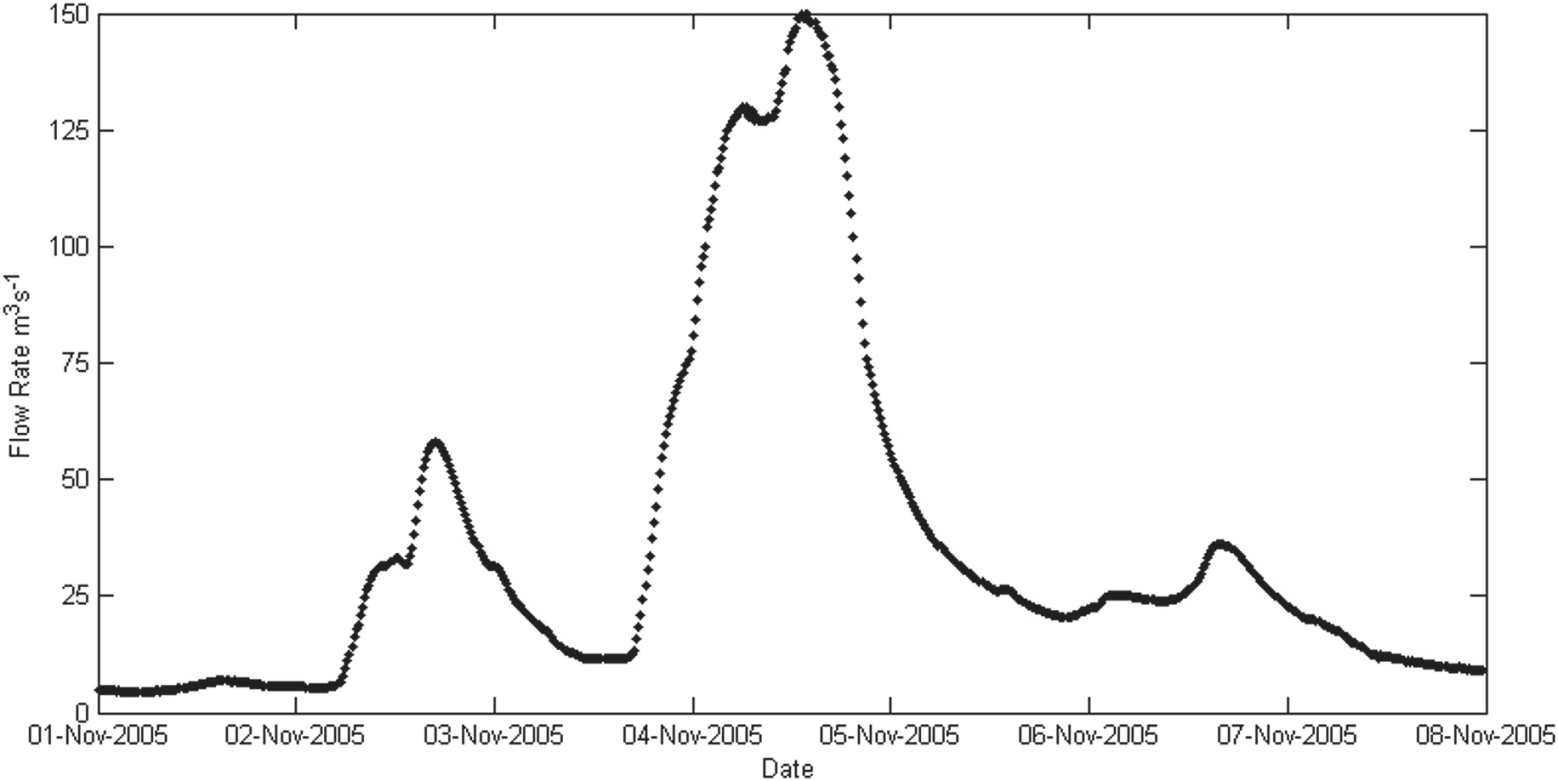
Example of a 1 in 50 year Hydrograph for the River Wyre, courtesy of the Environment Agency [39].

The Environment Agency provided a hydrograph of a specific high flow river event (maximum flow of 150 m^3^s^-1^) for the River Wyre, from gauging station 72002 Wyre at St. Michaels [39]. This event was selected by looking up suitable high flow events within the peaks over threshold (POT) file for the gauging station [40]. The flow data were provided at 15-minute intervals for the present-day extreme event, giving a statistically representative event hydrograph. The river was simulated by defining the section of the model boundary where the river enters the domain as a time varying water inflow that is forced by the hydrograph time varying flow data (Fig. 9). The EA also provided the river flow dataset at 15-minute intervals for the period 1976 to 2014. The flow peaks were extracted and a Weibull distribution was fitted which allowed the extrapolation of exceedance values to be calculated. The peak flow of the hydrograph represented a 1 in 50 year extreme river flow rate. Other return periods flow rates could be simulated by scaling the hydrograph to suit the relevant flow rate, in the same manner as the surge curve methodology proposed by the EA. It must be noted that the hydrograph is an example of an observed extreme event and not a statistical representation of all event curves.

### 2 Inundation Model

The freely available flood inundation model used here is LISFLOOD-FP [30]. LISFLOOD-FP is a 2D finite difference inundation model based upon the storage cell approach. This model was first formulated by Bates and De Roo (2000) [27] in order to provide a computationally efficient model capable of running on high-resolution LiDAR model domains. It alternatively allows an ensemble of model runs at lower resolution to allow probabilistic determination of variables (e.g. sea-level rise). LISFLOOD-FP has been continually developed since its inception, improving computational runtime and accuracy through inclusion of more realistic features [30].

LISFLOOD-FP has been used successfully in coastal applications, such as an assessment of the risk of future flooding in the Severn Estuary [41]. By using a probability distribution that is constructed to cover a range of future SLR scenarios by 2100 it was possible to assess the future flood risk to this area, also incorporating the low probability high impact scenarios. Including these low probability scenarios has a significant effect on the flood risk hazard for this study it was found to increase by 29.7% [41]. LISFLOOD-FP has also been used as an inundation model in the Solent, UK [42]. The methodology integrated a number of existing approaches for modelling floodplain inundations, including both over topping and defence breaching. The study also noted that the development of practical methods for the application of wave overtopping to the boundaries of coastal inundation models is an important area of future research [42]. LISFLOOD-FP has also been used in the Thames estuary for a different approach that simulates extreme sea-level scenarios that could potentially occur if the collapse of the Western Antarctic Ice Sheet (WAIS) occurs [43]. This study combined extreme SLR values that could result from this event with a 1 in 1000 year storm surge. It was found that a new Thames barrier would provide resilience to unexpectedly high SLR because of the large flood storage capacity the barrier would provide [43].

LISFLOOD-FP’s ability to explore coastal flooding has been tested on multiple occasions. On one of the occasions the model was tested against a major flooding event that occurred in 1981 [29]. The goodness of fit between observed and predicted inundation extent is quantified by comparing the sets of image pixels observed to be inundated and predicted to be inundated. Where the pixels overlap and are both inundated this value is equal to 1, if one of the sets of pixels is different to the other then this value is equal to 0 [44]. Pixel assessment across the whole domain allows the percentage of cells that coincide with each other to be measured giving a goodness of fit score [44]. In the above case the model scored a goodness of fit for the flood extent of 0.85 (85%), which given the uncertainty in deriving the observed extent is believed to be a good fit [29].

The key requirements for LISFLOOD-FP to ensure a good model simulation are an accurate (digital terrain model) DTM, boundary forcing (combination of tidal curve, surge curve and a sea-level rise parameter see section 1.1 for more details) and an estimation of the floodplain friction parameters. The DTM data were provided by the EA Geomatics department and consist of a LiDAR survey [38]. The data have a 1 m horizontal resolution and 0.05 m to 0.15 m vertical accuracy [38]. To reduce computational cost, the LiDAR data were re-sampled to 5 m horizontal resolution, this reduced the computation time to reasonable levels while maintaining enough detail to resolve urban features such as buildings and roads adequately. Re-sampling did affect the sea defences by smoothing the crest heights. These were digitised back into the raster DTM using a dataset provided by the EA [36]. The defence shape file provided the crest heights which were extracted, converted into a raster and mosaicked into the DTM raster file, other topographical features that needed to be removed (such as bridges, which act as artificial dams) or added in (for example lock gates) were digitised into the raster using a similar process. The final grid used within the model was 5 m horizontally with an accuracy of 0.15 m vertically.

### 3 Combining with Depth Damage Curves

To place an economic value on the simulated scenario inundation maps, the model cells in each simulation with depths greater than 0.05 m are combined with salt water depth damage curves [45]. Water depths less than 0.05 m are not considered to be damaging as they are below the vertical accuracy of the LiDAR used for the model domain. Salt water causes more damage than fresh and this is reflected in the depth damage curves. Using these curves the total cost (£M) for each scenario flood event can be calculated. Depth damage curves also vary depending on the land use of the cell inundated, e.g. arable, domestic housing, commercial etc. (see Tables 2 and 3) and the type of flood water and flood duration. Flood duration is difficult to quantify in these simulations as culverts and drainage measures are not resolved correctly by the 5 m grid. However, the depth damage curves can be selected for different flood durations. With the storm surge lasting for only 44 hours with a significant portion of it occurring during low tide, the short flood duration curves were used. Arable land-use was classified using the land cover 2007 dataset [46]. The different cost per hectare categories in Table 3 was based on Penning-Roswell et al. (2013) [45]. Urban, Sea bed, River channel and Salt marsh areas were classified using an Ordnance Survey topography layer [47]. This layer is a highly detailed view of the landscape which resolves individual buildings, roads and arable areas.

**Table 2:** Salt Water Depth Damage data for Housing, Road and Industrial flood inundation cells [45].

Housing Damage Curve		Road Curve		Industrial Curve	
depth(m)	cost per flood cell inundated (£)	depth(m)	cost per flood cell inundated (£)	depth(m)	cost per flood cell inundated (£)
**0**	£0	0	£0.00	0	£0.00
**0.05**	£3,317	0.25	£9.00	0.25	£1,475.00
**0.1**	£5,334	0.5	£12.00	0.5	£2,375.00
**0.2**	£9,109	0.75	£15.00	0.75	£3,075.00
**0.3**	£11,120	1	£19.00	1	£3,825.00
**0.6**	£13,525	1.25	£23.00	1.25	£4,425.00
**0.9**	£14,676	1.5	£29.00	1.5	£5,075.00
**1.2**	£16,084	1.75	£36.00	1.75	£5,750.00
**1.5**	£17,383	2	£49.00	2	£6,675.00
**1.8**	£18,869	2.25	£79.00	2.25	£7,300.00
**2.1**	£20,134	2.5	£92.00	2.5	£7,875.00
**2.4**	£21,325	2.75	£98.00	2.75	£8,325.00
**2.7**	£24,093	3	£110.00	3	£8,825.00
**3**	£25,308	-	-	-	-

**Table 3:** Inundation cost data for arable flood inundation cells [45].

Arable Costs	Cost per Hectare per Inundation Event (£)
**Arable and Horticulture**	1,150.00
**Improved Grassland**	180.00
**Rough Grassland**	50.00
**Neutral Grassland**	100.00

### 4 Surge Curve Sensitivity Analysis

Sensitivity analysis on the coincidence of the tidal high water and peak in surge elevation is strongly recommended by the methodology published by the EA [20]. The procedure followed was to run the flood simulation with the surge curve and tidal high water coinciding, then re-run with the peak surge elevation firstly occurring 2 hours before high tide, and secondly with the peak surge elevation occurring 2 hours after high tide. Comparing the flood extents, volumes and standard deviation of flood depths will show the sensitivity to the relative timing of the two peaks. Table 4 shows the output from these three simulations. With an offset of 4 hours (2 hours either side of high tide) the flood extent was found to not change by an area larger than 1000 m^2^, the volume varied by a maximum of 30,000 m^3^, due to an average depth increase of 0.01 m and the standard deviation by less than 0.01 m. From these results it has been assumed that the simulations are insensitive to surge and tide peak timing. This however only applies to this surge and tidal combination for this domain.

**Table 4:** Inundation statistics for different tide and surge offset simulations.

Model Run	Area of Land Inundated (Mm^2^)	Volume of Flood Water (Mm^3^)	Average Depth (m)	Standard Deviation of Depth
**Storm Tide Only Scenario**	4.26	4.61	1.08	0.96
**Surge peak 2hrs before tide**	4.26	4.63	1.09	0.96
**Surge peak 2hrs after tide**	4.26	4.64	1.09	0.96

### 5 Manning’s Friction Parameter *n*

**Fig 10:**
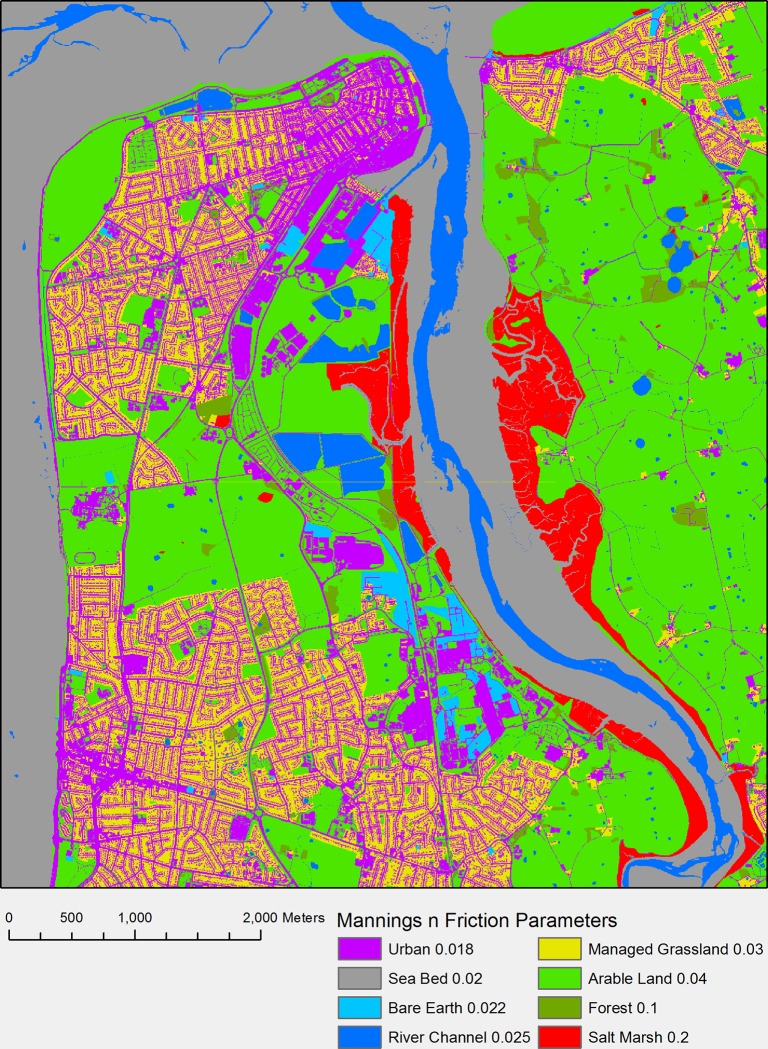
Mannings Friction n values for the model domain [54,55].

The inundation model requires a friction parameter also known as a manning value *n*, this can be a global value so every cell in the model domain has the same value or varied on a cell by cell basis. For these simulations a cell by cell varied approach was used. This was achieved by using the Ordnance Survey topography layer detailed in section 3 [47]. It was used to produce a grid (Fig. 10) that had variable friction values depending on the land use. The values shown in Fig. 10 were taken from Purvis et al, 2008 and Burke and Stolzenbach, 1983. Fig. 10 shows the friction values used for each land-use type.

## Results

**Fig 11:**
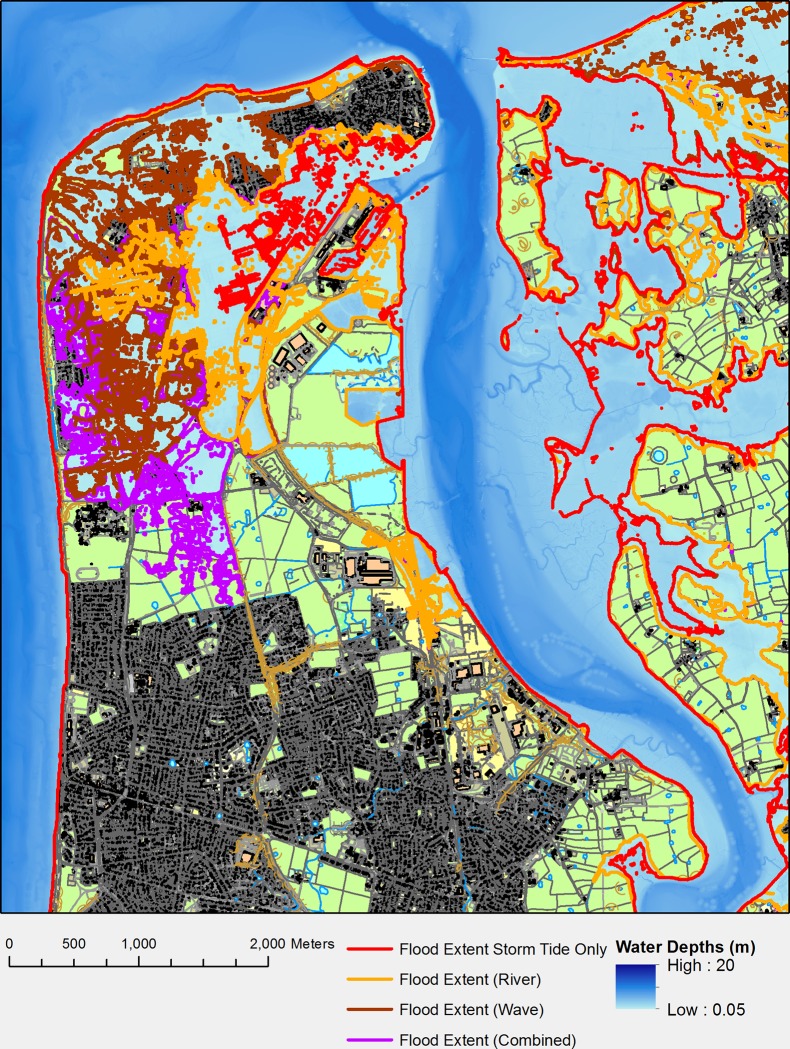
Fleetwood Flood Scenarios. Each line shows the flood extent due to a 1 in 250 year storm tide with 0.65 m SLR in isolation (red), with the addition of river flow (orange), wave overtopping (brown) and both waves and river (purple) [47].

The outputs from each simulations detailed in Fig. 1 have been displayed over Ordnance Survey topography layer data. The flood extent only shows water depths above 0.05 m as this is below the vertical accuracy of the LiDAR data and a 0.05 m flood depth adds little to the overall cost. Fig. 11 shows the flood at its maximum extent during the four simulations. All calculated areas, volumes, cost etc. apply to the peak of the storm tide event.

The simulation that comprised a storm tide and SLR, flooded 4.26 Mm^2^ of the land area, with a volume of 4.6 Mm^3^ of flood water. When the high flow river event was added, this increased to 9.68 Mm^2^ in area and 12.2 Mm^3^ of water. Removing the river flow and adding 1 in 100 year wave overtopping to the northern defences (the only defences that will overtop in this scenario), covers 8.33 Mm^2^ of the land with a volume of 6.99 Mm^3^ (Table 5). Fig. 11 shows these inundation projections varying spatially with the high flow river events increasing the inundation extent and depth on the arable east side of the river when compared with wave overtopping. The wave event causes a greater inundation of the urban areas on the west side of the river but with shallower depths on the east side over farm land when compared with the river event. Combining the two events, gives 12.75 Mm^2^ of inundation (Table 5) of the defined land area with a total volume of 14.04 Mm^3^.

**Table 5:** Output statistics for different projected inundation scenarios for a 1 in 250 year storm tide with 0.65m of SLR and additional flood factors.

Additional Factor	Area of Land Inundated (Mm^2^)	Volume of Flood Water (Mm^3^)	Average Depth (m)	Standard Deviation of Depth	Total Cost(£M)
**None**	4.26	4.6	1.08	0.96	48.87
**River forcing**	9.68	12.2	1.26	1.01	247.32
**Wave overtopping**	8.33	6.99	0.84	0.82	224.61
**Wave and River**	12.75	14.04	1.10	0.97	377.11

The inundated cells have been combined with depth damage curves from section 5 for their relevant land-use classification; these are 4 types of arable land (Table 3), residential buildings, roads, and industrial buildings. Table 6 shows that adding a high river flow event to a storm tide and SLR scenario has an impact of £198.4M increase in cost. Similarly adding wave overtopping creates a cost increase of £175.7M. Combining both forcing together generates an increase of £328.2M.

**Table 6:** Projected costs for each projected inundation scenarios for a 1 in 250 year storm tide with 0.65 m of SLR and additional flood factors.

Additional Factors	Arable Land Cost (£M)	Residential Housing Cost (£M)	Road Cost (£M)	Industrial Cost (£M)	Total Costs (£M)
**None**	0.16	47.68	0.23	0.81	48.88
**River forcing**	0.26	241.19	0.80	5.07	247.32
**Wave overtopping**	0.19	218.47	0.62	5.33	224.61
**Wave and River**	0.28	367.27	1.11	8.44	377.11

Table 7 shows the factor increases in area, volume and cost relative to the storm tide with SLR only scenario. Table 7 shows that adding a high river flow event to a storm tide and SLR scenario has a factor 2.3 increase in extent but a factor of 5.0 increase in economic cost. Similarly adding wave overtopping has a factor 2.0 increase in extent but a factor 4.6 increase in cost. Combining both forcings together has a factor of 3.0 increase in extent with a 7.7 increase in cost. This indicates that the addition of wave or river forcings causes non-linear increases in cost and extent. Table 5 has shown that the factor increase in area and volume is not linear when the two additional forcings are combined together; the same also applies to cost (Table 6). It is also clear that using flood extent area as a measure of a flood’s impact on a community significantly underestimates the economic impact of the flood. It is shown that the cost increases much more rapidly than the extent/volume of the flood water with more flood sources. It can be seen (Table 7) that when using flood extent or area as an indication of impact and not a full spatial depth damage curve cost analysis, under-estimates the economic cost by a factor of 2.2 for the high river flow scenario, a factor of 2.4 for the wave overtopping, and a factor of 2.6 for the combined scenario.

**Table 7:** Comparison of changes in extent and volume and cost for a 1 in 250 year storm tide with 0.65 m of SLR and additional flood factors in comparison to the storm tide with SLR alone.

Additional factors	Factor increase in Area	Factor increase in Volume	Factor increase in cost
**River flow**	2.27	2.65	5.06
**Wave overtopping**	1.95	1.50	4.60
**Combined**	2.99	3.05	7.71

Table 8 shows the mean depth with associated standard deviation, the average depth column in Table 8 varies from 0.84 m (wave overtopping) to 1.26 m (river forcing). The wave and river scenario has an average water depth of 1.1 m. This is due to the wave scenario having a larger area of relatively low depths which can be quantified by the difference in area compared with the difference in volume for the two scenarios. The river scenario has an increase in inundation volume of 74.5%, compared with only an increase of 16.2% in inundation area. This means that the river is causing more damage (due to deeper depths) in a smaller urban area while the waves cause less damage (due to shallower depths) but over a bigger urban area.

**Table 8:** Number of building cells inundated with average depth.

Additional factors	Number of Building Cells inundated	Mean Depth (m)	Standard Deviation of Depth
**River forcing**	20238	0.67	0.49
**Wave overtopping**	22461	0.42	0.34
**Combined**	32885	0.60	0.46

Waves, while having a lower total economic impact, are likely to have a bigger impact on the community as more buildings are inundated. In the river scenario 20238 grid cells categorised as a residential building (approx. 6746 homes) have been inundated at an average depth of 0.67 m, (Table 5) whereas the wave scenario has 22461 building grid cells (approx. 7487 homes) are inundated at an average depth of 0.42 m (Table 5). Combining the river and wave scenarios results in 32885 building grid cells (approximately 10,961 houses) being inundated at a mean depth of 0.60 m (Table 5).

**Fig 12:**
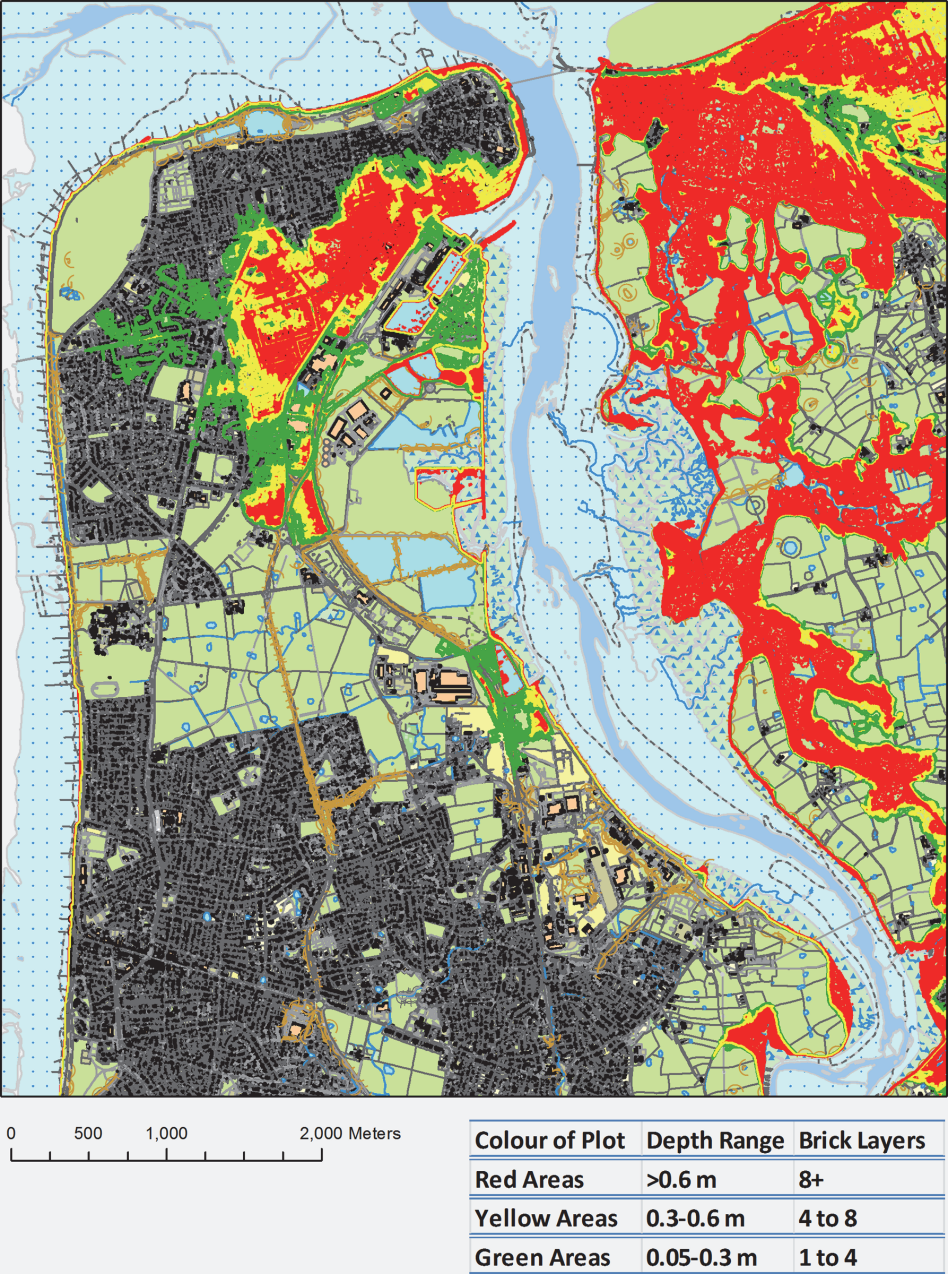
Example Brick Course Map for the river scenario, 1 in 250 year storm tide, 0.65 m SLR, green areas have flood depths of up to 4 brick courses, yellow areas have flood depths of 4 to 8 brick courses and flood depths of greater than 8 brick courses are in red [47].

**Fig 13:**
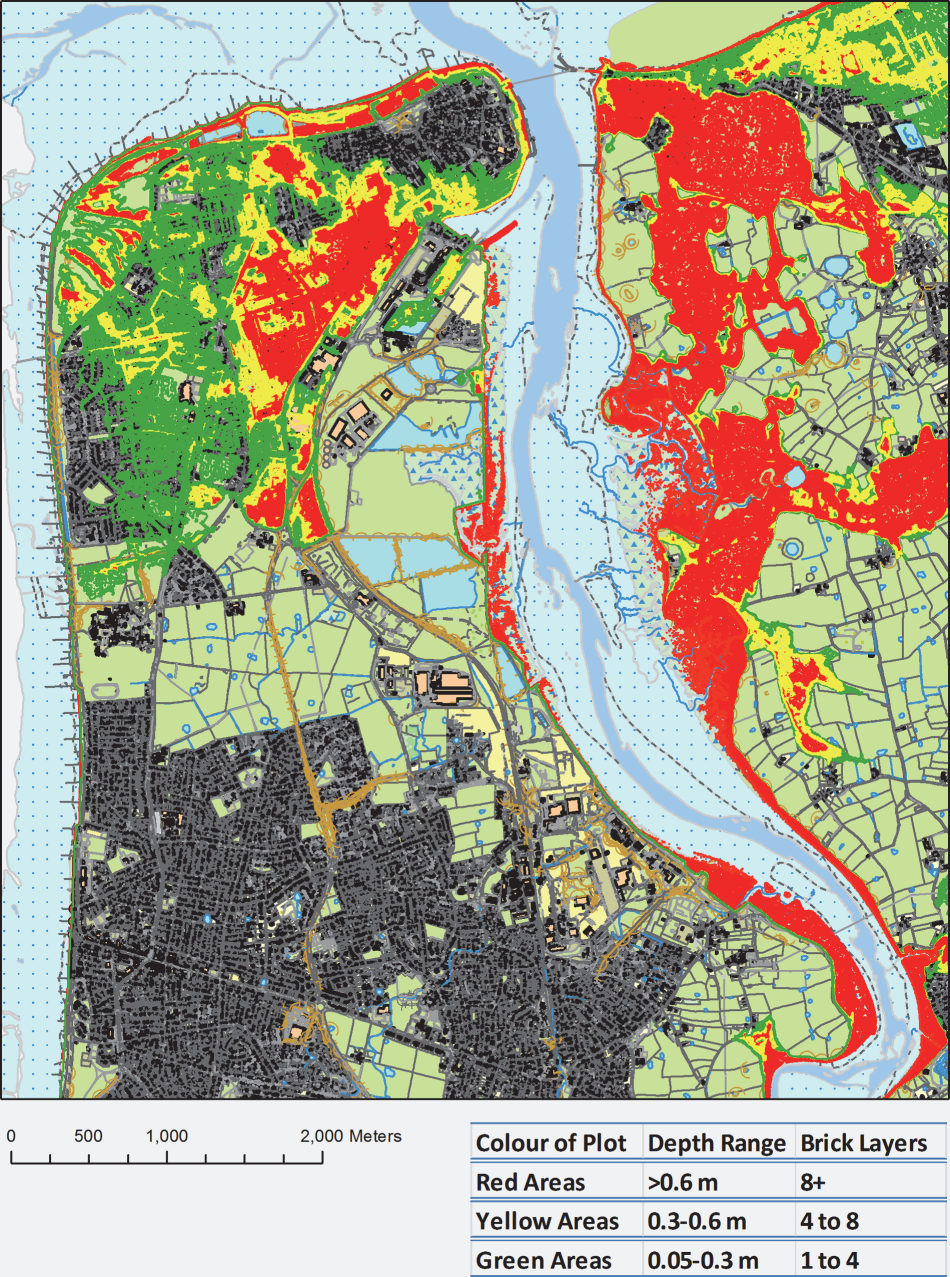
Example Brick Course Map for the wave overtopping scenario, 1 in 250 year storm tide, 0.65 m SLR, green areas have flood depths of up to 4 brick courses, yellow areas have flood depths of 4 to 8 brick courses and flood depths of greater than 8 brick courses are in red [47].

**Fig 14:**
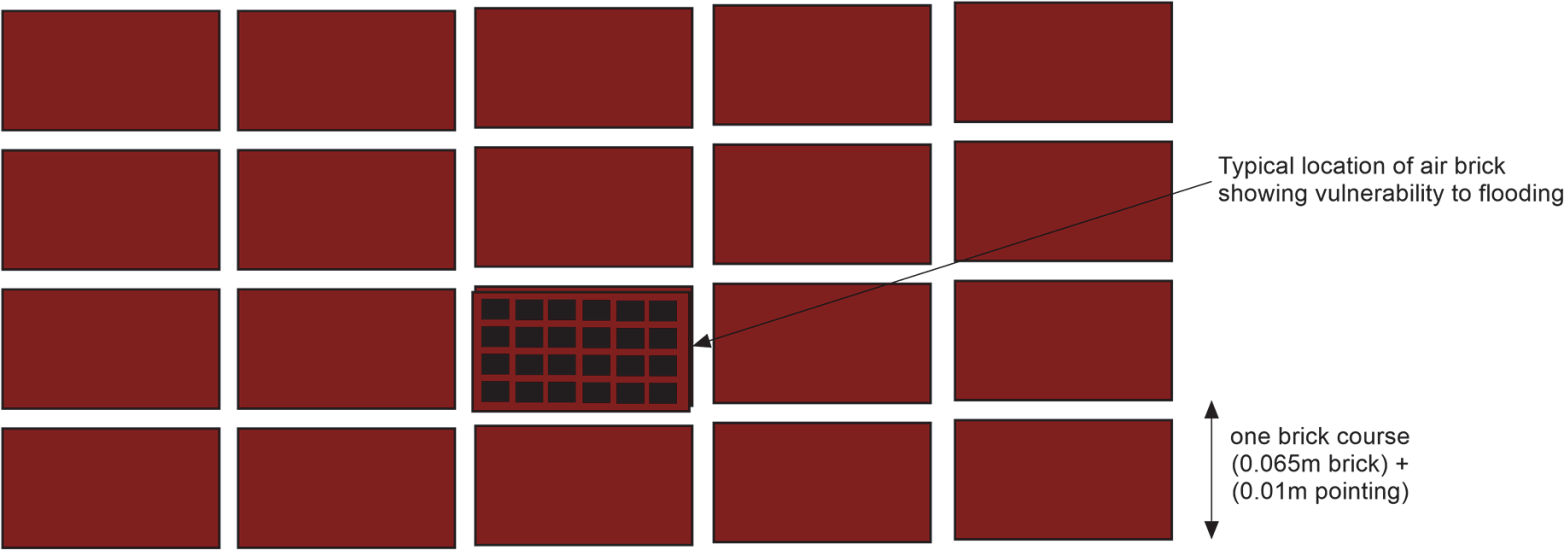
Brick course schematic.

Figs. 12 and 13 consist of mitigation maps presented as brick course layer maps for both the wave overtopping and river forcing scenario. Brick courses have been defined as a single horizontal layer of bricks in a building which allows a practical way of seeing the impact of the flooding. A brick course has been based on the thickness of a standard brick (0.065 m) and one pointing layer (0.01 m) giving a total height of 0.075 m (Fig. 14) [48]. To create these brick course maps, the flood depths have been converted into a contour plot contoured at 0.075 m intervals which have been coloured as follows, green contours showing areas with water depths up 4 brick courses, yellow contours showing areas with water depths between 4 and 8 brick courses and red contours showing areas with water depths greater than 8 brick courses.

Across all scenarios the worst affected region within Fleetwood is a small area that experiences greater water depths; it is highlighted red area on the west side of the river in both Figs. 12 and 13. This area would be a significant hazard during the projected flood events with depths of up to 2.25 m realised. Outside of Fleetwood, the community on the east side of the river that is built along the coastline also experiences significant water depths, especially during the wave overtopping scenario, where the whole area is highlighted red. The red areas on the east side of the river are predominantly salt marshes and arable land which is not significantly affected by short duration flood events.

## Discussion

The scenario flood projections cannot be taken as a prediction of a future event; they are an extreme joint scenario of events with projected SLR. The SLR values used are at the lower probability end of the UKCP09 projections [15], but nevertheless they are still potential outcomes in future climate. Especially if events like the WAIS collapse occur as high levels of SLR are projected as a response to these events.

**Fig 15:**
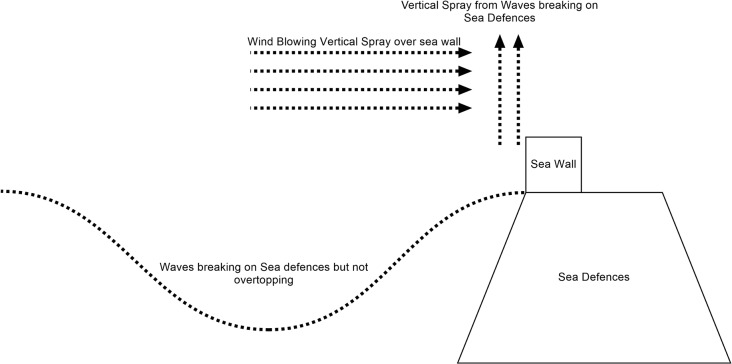
Diagram showing how vertical spray from waves breaking on sea defences can be blown as spray over the top of the defences causing minor flooding.

The recent flooding event in Fleetwood on the 5th December 2013 caused a limited amount of flooding. After discussing the event with the local authority, it was found that water had got over the sea defences in the form of windblown spay from waves breaking on the sea defences (Fig. 15). This is something that currently cannot be added to the inundation model, but if a method could be found to simulate the spray being blown over the defences, then it could be added to the model in a similar manner as the wave overtopping simulations. This additional capability would be very useful when modelling storm surges like the one that occurred on the 5^th^ of December 2013.

Another factor to acknowledge is that because these events are low probability and represent long-term SLR, any long-term morphological changes that occur during this time period, such as change in the beach profiles and the feedback on tides and wave conditions, will not be accounted for in the methodology. The current shoreline management plan has a “hold the line” policy in this area so it is likely that new defences will be commissioned locally in the future which should be more resilient than the current ones [49]. Also it should be noted that the inundation model does not account for any changes in the beach profile that may occur in the future, which could affect the flooding in each scenario.

As per section 4, sensitivity analysis was performed on the storm tide flood extent over the Fleetwood domain in response to the timing of tidal high water relative to the surge curve peak. It was found that moving the surge peak 2 hours before and 2 hours after the tide peak slightly increased the average depths of inundation (Table 4). The differences in this instance were small (0.01 m), hence it has been assumed that the simulations are insensitive to the relative timing of the surge and tide. However, the analysis is still important as in other cases, with different storm tide, SLR, river and wave forcings, the difference could be significant.

This research shows that adding a high river flow event to a storm tide and SLR scenario increases the flood extent by a factor of 2.3, but increases the cost by a factor of 5. Similarly adding wave overtopping increases the flood extent by a factor of 2.0 but the cost by a factor of 4.6. Combining both forcings together increases the extent by a factor of 3.0 with a 7.7 factor increase in cost. Comparing the changes in extent and volume with the cost indicates that flood risk analysis must consider the economic costs for land usage.

There is significant overlap between the scenarios in Fig. 11 and it is clear that impacts from either event do not combine in a linear way when simulated together. It is also clear from the breakdown of costs (Table 6) that the biggest factor in the make-up of total flooding costs is the cost of residential housing; this is because residential houses have significant costs in clean up and repair [45].

To try and mitigate flooding clean up and repair costs to houses the brick course maps (Figs. 12 and 13) can be utilised. They are maps showing green, yellow and red areas that have been flooded. The green and yellow areas are flood water depths under 8 brick courses, this flood water depth is a significant threshold as houses can be made resilient up to these flood depths. However, for flood depths over 8 brick courses, shown in the red areas of the brick course maps (Figs. 12 and 13), the flood water is likely to cause structural damage if it is unable to enter a property.

As mentioned in the previous paragraph, under 0.6 m water elevation (or 8 brick courses), houses can be made resilient to flooding [50] flood mitigation measures can be added so the building is able to withstand these flood depths [51]. These measures can take the form of periscope air bricks, having the front door elevated requiring steps or a ramp for access and/or a properly fitted cover for the doors of the house which is superior to using sandbags, etc. Recent research carried out by CIRIA (Construction Industry Research and Information Association), the Department for Communities and Local Government (DCLG) and the Environment Agency recommend that for new builds the use of resistance measures should be limited to flood depths of 0.6 m predominantly because the structural integrity of a standard building may be comprised above this level [51]. The possibility of stressing of masonry that could induce cracks and leaks is greatly increased above this level [50]. At depths of 0.9 m or greater there is also the risk from large floating debris such as tree trunks and vehicles [50]. Accredited temporary resistance products which cover apertures in buildings have design protection depths of up to 0.9 m and it should be noted that the depth of which structural integrity of buildings is comprised by flood waters is still an active area of research [50].

From the brick course map in Fig. 13, it can be seen that it is possible to lessen the impact of the wave scenario in this case study, as the majority of homes at risk will experience less than the flooding threshold for flood resilience measures to be effective. Making the houses resilient from the river scenario (Fig. 12) will be more costly as it will require river defences and increased river flood storage capacity to keep the flood waters away from the houses or at least mitigate the depth of the flooding to under the threshold for effective flood mitigation measures.

Risk in £yr^-1^ to the coastal communities is notably missing from the results. This is due to the difficulty in calculating the probability of these scenarios occurring. It is plausible for these scenarios to occur at some point in the future, but they are very unlikely to occur in any given year. While the probabilities of each of the events that make up the scenario have been calculated, it is difficult to combine them together into one. This is because the events are not completely independent of each other requiring multivariate analysis to be performed. This is beyond the scope of this paper.

The duration of the flood on a grid cell by grid cell basis is difficult to determine. Although the inundation model is capable of simulating it, the domain is not sufficiently high enough in detail to resolve all the drainage channels that would be present on the sub-5 m scale. Also the model would not be able to take into account any pumping work that could be carried out or the drainage provided by any sluice gates. The result from this is that duration times for the drying sections of the simulation are over estimated and cannot be relied on. This could be improved by running the simulations at a higher resolution but comes at the cost of significantly increased computation time. Being able to define flood water duration for each grid cell in addition to this research would be useful as it would show how long flood waters stay in grid cells, giving coastal managers more information to allow better emergency response planning.

The inundation model also has the ability to output velocities from the simulations, these can be combined with flood depths to generate a flood hazard rating [52]. This can be used as part of emergency planning as it will show areas that have a significant hazard to life, values less than 0.75 are considered low hazard, values between 0.75 and 1.25 are moderate hazard, 1.25 and 2.0 are significant hazard and values greater than 2.0 are considered extreme hazard. Even emergency services should not go into areas with a hazard rating of 2 or above [52]. Being able to assign these ratings spatially, identifying the highest risk areas would be a significant benefit to coastal communities and coastal managers. Providing information on impacts in areas of high flood risk would allow coastal authorities to warn people living in high risk areas and try to reduce the risk by deploying resilience measures to reduce flood depth heights and velocities.

Being able to calculate the risk in £yr^-1^ for the various scenarios, would be useful in cost-benefit assessments to calculate the most efficient investment options. Examples of these options include:

- Increasing sea defence crest height
- Investing in flood defence mitigation measures at individual house level
- Increasing river flood storage capacity
- Increasing sea defence width to allow height to be increased if needed.

As well as calculating the most efficient investment options, future work could also include deciding the best time to deploy these options and where, for example, which sections of defence to increase in height rather than all of them or which houses would benefit most from resilience measures and when depending on the current level and rate of SLR.

The implications of this work is that areas could be more susceptible to flooding than previous flood inundation models demonstrate as the combination of waves and rivers significantly increases the impact of flood events. This has implications for any community that is located in an area that can be affected by large waves and high river flows.

## Conclusions

We have demonstrated a method to combine accessible physical models to incorporate multiple flood forcing to simulate inundation in coastal flood risk regions where terrain data is available. Using Fleetwood as a case study, a technique to convert the simulated inundation depths into maps of economic impact is demonstrated. Such a conversion is not only applicable to coastal communities but could also be applied to regions at risk of fluvial and/or pluvial flooding. Mapping the economic costs in this way helps to inform flood risk management, providing information to investigate the cost benefit analysis of adaptation plans to build more resilient communities to future flood risk. The brick course maps have demonstrated a new representation of flood maps that can be easily disseminated to stakeholders and improve engagement.

This study has identified that relying on still water flood simulations can under estimate the economic impact of a plausible future flood event by up to a factor of 7.7. With the addition of other potential flood forcings (in this case wave overtopping and high river flow events), measured parameters are significantly increased creating a much larger impact than if a still water level only scenario was used.

Additionally, using increases in physical parameters to measure the increase in flood impact of an event can lead to under-estimation. In the scenario with additional wave and river forcing, the economic impact increases by a factor of 7.7 over the storm tide only event, where the flood extent only increases by a factor of 2.99.

It has also been shown that the impact from one or the other additional forcings is not linear when combined together. This is illustrated by wave overtopping causing an economic factor increase of 4.6 against the storm tide and SLR only scenario, the high flow river event causing an increase of 5.1 respectively. When these forcings are combined, the factor increase is 7.7 which is less than the sum of the two separate economic impacts of 9.7.

Finally, it has been found in the case of Fleetwood that the main fraction of economic cost is due to residential housing. Mitigation measures that combat the impacts to residential housing should therefore be made a priority in any resilience planning or investment decisions.

In a wider context this paper shows that when performing flood risk assessments of coastal floodplains it is important to consider additional flood sources, such as wave overtopping in areas with significant wave action, and river flows in areas susceptible to river floods, or in the case of Fleetwood both of these factors. A suitable place to perform this methodology is along the North Wales coastline encompassing the towns of Prestatyn, Rhyl and Towyn and the River Clwyd. For flooding from river flow and storm tide coincidence, the Humber and Thames estuaries are good candidates for this analysis, in which case wave overtopping would be less of a concern due to the lower wave heights experienced in these estuaries.
